# The Mortality Risk and Pulmonary Fibrosis Investigated by Time-Resolved Fluorescence Spectroscopy from Plasma in COVID-19 Patients

**DOI:** 10.3390/jcm11175081

**Published:** 2022-08-30

**Authors:** Tomasz Wybranowski, Jerzy Pyskir, Maciej Bosek, Marta Napiórkowska, Michał Cyrankiewicz, Blanka Ziomkowska, Marta Pilaczyńska-Cemel, Małgorzata Pyskir, Milena Rogańska, Stefan Kruszewski, Grzegorz Przybylski

**Affiliations:** 1 Biophysics Department, Faculty of Pharmacy, Collegium Medicum in Bydgoszcz, Nicolaus Copernicus University in Toruń, 85-067 Bydgoszcz, Poland; 2 Department of Lung Diseases, Neoplasms and Tuberculosis, Faculty of Medicine, Collegium Medicum in Bydgoszcz, Nicolaus Copernicus University in Toruń, 85-067 Bydgoszcz, Poland; 3 Department of Rehabilitation, Faculty of Health Sciences, Collegium Medicum in Bydgoszcz, Nicolaus Copernicus University in Toruń, 85-067 Bydgoszcz, Poland

**Keywords:** collagen, COVID-19, fluorescence lifetime, pulmonary fibrosis

## Abstract

A method of rapidly pointing out the risk of developing persistent pulmonary fibrosis from a sample of blood is extraordinarily needed for diagnosis, prediction of death, and post-infection prognosis assessment. Collagen scar formation has been found to play an important role in the lung remodeling following SARS-CoV-2 infection. For this reason, the concentration of collagen degradation products in plasma may reflect the process of lung remodeling and determine the extent of fibrosis. According to our previously published results of an in vitro study, an increase in the concentration of type III collagen degradation products in plasma resulted in a decrease in the fluorescence lifetime of plasma at a wavelength of 450 nm. The aim of this study was to use time-resolved fluorescence spectroscopy to assess pulmonary fibrosis, and to find out if the lifetime of plasma fluorescence is shortened in patients with COVID-19. The presented study is thus far the only one to explore the fluorescence lifetime of plasma in patients with COVID-19 and pulmonary fibrosis. The time-resolved spectrometer Life Spec II with the sub-nanosecond pulsed 360 nm EPLED® diode was used in order to measure the fluorescence lifetime of plasma. The survival analysis showed that COVID-19 mortality was associated with a decreased mean fluorescence lifetime of plasma. The AUC of mean fluorescence lifetime in predicting death was 0.853 (95% CI 0.735–0.972, *p* < 0.001) with a cut-off value of 7 ns, and with 62% sensitivity and 100% specificity. We observed a significant decrease in the mean fluorescence lifetime in COVID-19 non-survivors (*p* < 0.001), in bacterial pneumonia patients without COVID-19 (*p* < 0.001), and in patients diagnosed with idiopathic pulmonary fibrosis (*p* < 0.001), relative to healthy subjects. Furthermore, these results suggest that the development of pulmonary fibrosis may be a real and serious problem in former COVID-19 patients in the future. A reduction in the mean fluorescence lifetime of plasma was observed in many patients 6 months after discharge. On the basis of these data, it can be concluded that a decrease in the mean fluorescence lifetime of plasma at 450 nm may be a risk factor for mortality, and probably also for pulmonary fibrosis in hospitalized COVID-19 patients.

## 1. Introduction

An outbreak of the novel coronavirus nCoV-19 (SARS-CoV-2), responsible for the coronavirus disease-19 (COVID-19), was first reported in Hubei province, China, on 31 December 2019. It has rapidly spread globally, with approximately 3 million confirmed infections and 200,000 deaths within the first four months [1]. SARS-CoV-2 primarily affects the respiratory system. Clinical, radiographic, and autopsy reports of pulmonary fibrosis were commonplace following SARS and MERS. Current evidence suggests that pulmonary fibrosis could complicate infection by SARS-CoV-2 [2]. Pulmonary fibrosis (PF) is also a known sequela of severe and/or persistent damage to the lung from other causes, such as connective tissue disorders, chronic granulomatous diseases, medications, and respiratory infections [3]. Fibrosis could be viewed as a consequence of a disordered wound healing process, and may be directly related to the severity of an inciting event [4,5]. Various mechanisms of lung injury in COVID-19 have been described, with both viral and immune-mediated mechanisms being implicated [6]. Apart from these, additional factors could predispose individuals to severe lung injury, and lead to an increased risk of mortality or pulmonary fibrosis in survivors. The symptoms associated with COVID-19 range from mild upper respiratory tract involvement to severe acute respiratory distress syndrome (ARDS), requiring long-term oxygen therapy due to pulmonary fibrosis [7,8].

Since 7.2–31% of COVID-19 patients suffer from virus-induced ARDS [9], PF can be considered an important long-term adverse health outcome of the disease [10]. Indeed, bilateral, extensive lung involvement and histopathological findings of diffuse alveolar damage and fibromyxoid cell exudates have been considered structural factors that predispose patients, who have overcome a severe case of COVID-19, to impaired lung reconstruction [11]. Of interest, patients with severe COVID-19 leading to ARDS may also show full thickness tracheal/large airway cartilage lesions with fibrous-hyaline degeneration; this pathologic feature is uncommon in individuals with non-COVID-19 ARDS, and may have contributed to the unexpectedly high prevalence of pneumomediastinum reported during the second wave of the COVID-19 pandemic [12,13]. The major morbidity and mortality from COVID-19 is due to acute viral pneumonitis that evolves into ARDS. Furthermore, COVID-19 patients may be affected by extra respiratory involvement. Up to 20% of COVID-19 patients develop acute respiratory failure (ARF) and ARDS, and require oxygen therapy delivered through a high-flow nasal cannula (HFNC), non-invasive ventilation (NIV), and invasive mechanical ventilation (IMV) [14,15,16,17]. One of the main characteristics of COVID-19 pneumonia is a dissociation between the severity of the hypoxemia and the relatively preserved respiratory mechanics [18]. Han and co-authors recently reported that fibrotic-like changes observed with computed tomography (CT) performed at 6 months during follow-up persist in approximately one-third of patients with COVID-19 [19].

As progressive pulmonary fibrosis is one of the possible consequences of COVID-19 pneumonia, there is a great need for a new blood biomarker to detect it. Pulmonary fibrosis is characterized by excessive deposition and degradation of collagen in the tissue. Thus, most markers of pulmonary fibrosis are related to the search for proteolytically cleaved matrix degradation fragments, or neoepitopes, which are released into the blood circulation. Enzyme-linked immunosorbent assays (ELISA) are often used for the detection of various types of collagens. Some collagen-degrading matrix metalloproteinases (MMPs) are also considered markers of fibrosis [20,21,22]. The contents of glycine, proline, and hydroxyproline are often estimated due to their presence only in collagen. However, the above measurements are extremely time-consuming and very expensive, so it is difficult to apply them in a hospital setting.

There is still a great need for easy diagnostic methods that reveal strong predictors of mortality, and those that allow the assessment of the patient’s condition and post-infection fibrosis. Fluorescence spectroscopy of whole blood or its components has been a very convenient tool in the diagnoses of many diseases [23]. The method enables quick investigations of biological samples, and requires no additional reagents. Fluorescence lifetime is a measure of excited-state stability that depends on the local environment of the fluorophore; it may vary with, for example, conformational changes in molecules, and during molecular interactions with other molecules. On the other hand, it does not depend directly on the concentration of the fluorophore, so unlike measuring only the fluorescence intensity, it is applicable in systems where the exact concentration is unknown or impossible to control [24]. Measurement of the fluorescence lifetime using the time-correlated single photon counting (TCSPC) method is based on the assumption that the statistical distribution of time intervals between excitation and emission obtained for individual fluorophores is asymptotically (after a large number of excitation–emission cycles) consistent with the distribution of the fluorescence lifetime of a large number of fluorophores excited at once. Each TCSPC cycle begins with a sub-nanosecond excitation laser pulse, and ends with the detection of a single photon. The time between these events is accurately measured. The histogram of the obtained intervals reflects the fluorescence decay of the entire sample, and is analysed by fitting the multi-exponential function to extract the amplitude and lifetime of the fluorescence of different types of fluorophores [25,26].

The formation of excessive scar tissue in the lungs in COVID-19 may be associated with a higher risk of mortality. Meanwhile, an increase in the concentration of collagen degradation products in blood circulation during the process of lung remodeling in the early phase of wound healing may determine the extent of pulmonary fibrosis. The results of our previous study revealed that an increase in the concentration of hydrolysed collagen added to plasma reduced the mean fluorescence lifetime at 450 nm [27]. Moreover, the decrease in mean fluorescence lifetime was dependent on the degree of collagen hydrolysis. Thus, we hypothesize that the measurement of the fluorescence lifetime of plasma provides information about pulmonary fibrosis in COVID-19 patients. To the best of our knowledge, there are no other compounds emitting at 450 nm in blood plasma that are related to COVID-19 survival and mortality that could shorten the fluorescence lifetime of plasma. Furthermore, in our other study, we showed that the plasma of patients with a potential high probability of developing left ventricular remodeling after acute myocardial infarction is characterised by a decrease in fluorescence lifetime [28]. We revealed a negative significant correlation (*p* < 0.001) between plasma fluorescence lifetime and brain natriuretic peptide that is secreted by the left ventricle in response to mechanical wall stress, and is involved in cardiac remodeling. To our knowledge, there are no reports of the use of time-resolved fluorescence spectroscopy in COVID-19 patients and, in general, in patients with pulmonary fibrosis. 

## 2. Materials and Methods

### 2.1. General Characteristics of the Study Patients 

In this study, we included patients (Table 1) hospitalized in the Department of Lung Diseases, Neoplasms and Tuberculosis at the Regional Center of Pulmonology in Bydgoszcz, Poland, from April to December 2021. Patients admitted to the hospital department who were 18 years of age or older (with no upper age limit), and who had been hospitalized with COVID-19 pneumonia that had been confirmed by a positive reverse transcription-polymerase chain reaction (RT-PCR) test result from a nasopharyngeal swab, according to the World Health Organization (WHO) criteria [29] and radiographic imaging (HRCTs were performed using a 64-slice Siemens Somatom Sensation (Siemens Healthcare, Erlangen, Germany) system with a slice thickness ≤ 0.5 mm) or chest X-ray, were eligible for enrolment. Patients had a blood oxygen saturation below 94% while breathing ambient air, but were excluded if they were receiving continuous positive airway pressure, bilevel positive airway pressure, or mechanical ventilation. The patients received standard care according to local practice, which could include antiviral treatment, the limited use of systemic glucocorticoids (recommended dose ≤ 1 mg per kilogram of body weight of methylprednisolone or equivalent), oxygen therapy, including high-flow nasal oxygen therapy, convalescent plasma, low-molecular-weight heparin, and supportive care.

All patients had undergone basic laboratory tests assessing the advancement of inflammation, the function of their liver and kidneys, and the parameters of their coagulation system. The mean percentage of lung involvement as assessed by the application of HRCT pneumonia analysis was 28%. The study group of COVID-19 patients was invited back for us to take another round of blood samples after 6 months. The reference group was 10 patients hospitalized in the pulmonological department from the same centre who were not treated for COVID-19. These patients were admitted urgently for bacterial pneumonia, after excluding SARS-CoV-2 infection. The next reference group was 9 patients with previously diagnosed idiopathic pulmonary fibrosis (IPF) that was identified in accordance with the 2018 recommendation of the scientific societies [30]. The control group consisted of 15 subjects who were considered healthy.

### 2.2. Sample Preparation 

A 4-millilitre blood sample was taken for examination from each subject included in the study. For each COVID-19 patient, the sample was drawn 2–4 days upon admission to hospital department. All samples were processed within 2 h of collection. Blood samples were added to standard sterile polystyrene tubes containing EDTA, and then centrifuged at 3500 rpm at 4 °C for 5 min to obtain plasma. The plasma fraction was collected and stored at −80 °C until measurement. Multiple freeze–thaw cycles were avoided. Measurements were taken within 1 h of defrosting the sample.

### 2.3. Time-Resolved Fluorescence Spectroscopy Measurements

A time-resolved spectrofluorometer Life Spec II (Edinburgh Instruments Ltd., Livingston, UK) with a sub-nanosecond pulsed EPLED^®^ diode emitting light of a 360-nanometre wavelength was used in order to measure the fluorescence lifetime of plasma. Plasma samples were not diluted. The exposure time of the samples was 5 min. Fluorescence measurements of the plasma were made at wavelengths of 450 nm. The samples were brought to room temperature prior to performing the assay. The measurements were carried out with the use of quartz 3.5 × 10 mm cuvettes. 

The fluorescence lifetimes were obtained by the deconvolution analysis of the data using the multiexponential model of fluorescence decay, and the instrument response function was taken into account. Then, the mean fluorescence lifetime value (mFLT) was calculated as the weighted average of fluorescence lifetimes obtained from the three-exponential model of fluorescence decay. As averaging weights, the contributions of individual components (areas under decay curves) to the total fluorescence were taken. The appropriate number of exponents was determined on the basis of chi-square (χ^2^) statistical analysis and the visual assessment of residual plots.

### 2.4. Statistical Analysis

The preliminary step of the statistical analysis was the Shapiro–Wilk test of the normality of the distribution of the measured parameters. If the variables were normally distributed, the differences between independent and dependent samples were compared with the Student’s *t*-test, and dependencies between variables were determined with Pearson correlation coefficients (r values). In case of the non-normally distributed variables, differences were compared with the Mann–Whitney U test and the Wilcoxon signed-rank test, respectively, and dependencies were determined with Spearman’s rank correlation coefficients. The probability of surviving to day 30 in COVID-19 patients was performed by using Kaplan–Meier curves, and tested with the log-rank test (Mantel–Haenszel). Differences were considered significant at *p* < 0.05. As a class predictor for the receiver operating characteristic (ROC) curve for a given parameter (mFLT), the value of this parameter was used.

## 3. Results

### 3.1. Analysis of mFLT in the Study Groups 

**Figure 1:**
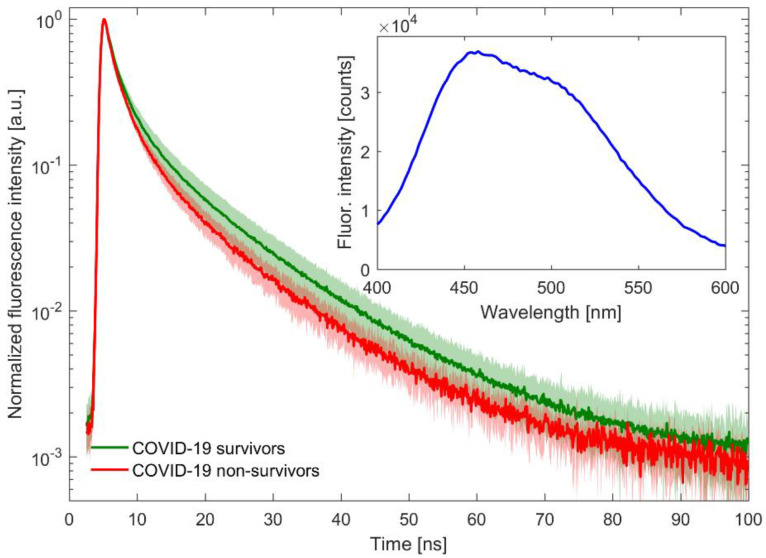
Fluorescence decay curves of plasma samples from patients who survived (green line) and did not survive (red line) COVID-19. Averaged normalized decay curves with corresponding one-standard-deviation bands are presented on the main plot. The inset plot shows typical fluorescence spectra of examined samples.

As shown in Figure 1 (inset plot), plasma excited by light pulses of the 360-nanometre laser diode exhibits the most intense emission near 450 nm, thus changes in fluorescence in this region are of interest in this study. The research focuses only on the recording and analysis of fluorescence decay curves, because the lifetime of fluorescence, unlike its intensity, is insensitive to, for example, scattering due to sample turbidity.

The raw data obtained from the TCSPC method have a fairly large signal intensity spread. The same data after zero-max normalization clearly show faster fluorescence decay in the group of patients who did not survive COVID-19, compared to the patients who recovered (see the main plot in Figure 1). This observation prompted the authors to carry out an in-depth analysis, in particular, to compare the COVID-19 group with healthy patients and with those suffering from other lung diseases.

**Figure 2:**
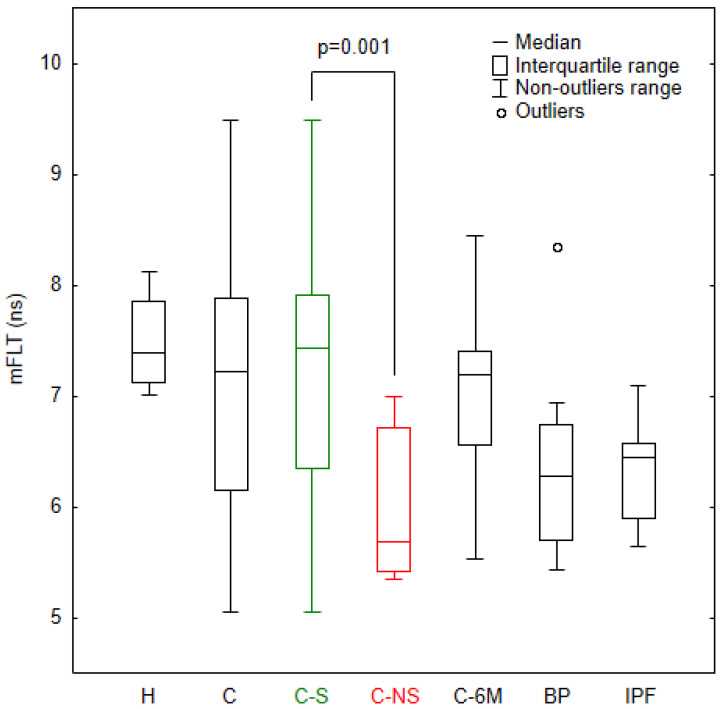
Mean fluorescence lifetime (mFLT) values for study groups. H—healthy, C—all COVID-19 patients, C-S—COVID-19 survivors, C-NS—COVID-19 non-survivors, C-6M—COVID-19 patients after 6 months from infection, BP—bacterial pneumonia patients without COVID-19, IPF—idiopathic pulmonary fibrosis patients. Data that were more than 1.5 interquartile range from this range were considered outliers.

In this study, the authors analysed only values of mFLT weighted by the fractional contribution of each component calculated from three-exponential models. Figure 2 shows that the mFLT values for the COVID-19 patients had a much more inhomogeneous distribution than those for the healthy subjects. In this light, it should be noted that the mean fluorescence lifetime for some COVID-19 patients was well below the range for healthy individuals. We observed a significant decrease in the mFLT in COVID-19 non-survivors (*p* < 0.001), in bacterial pneumonia patients without COVID-19 (*p* < 0.001), and in patients diagnosed with idiopathic pulmonary fibrosis (*p* < 0.001), relative to healthy subjects. Subgroup analyses revealed that the non-survivor group had a shorter fluorescence lifetime with a high level of significance (*p* = 0.001), relative to survivor patients. The 6-month post-hospitalized COVID-19 patients showed also a shorter mFLT compared to healthy subjects (*p* < 0.05).

### 3.2. Mortality Analysis

**Figure 3:**
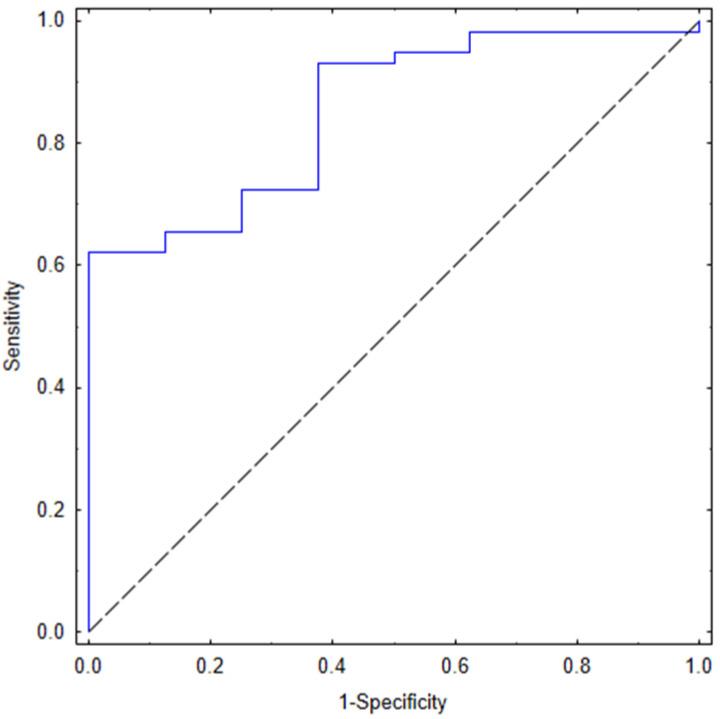
The ROC curve for mean fluorescence lifetime (mFLT) for COVID-19 survivors and non-survivors (C-S and C-NS in Figure 2, respectively).

The ROC curve analysis is shown in Figure 3, and indicates that the mFLT value (AUC = 0.853; 95% CI 0.735–0.972; *p* < 0.001; cut-off points = 7 ns; 62% sensitivity and 100% specificity) differentiated the groups very accurately. 

**Figure 4:**
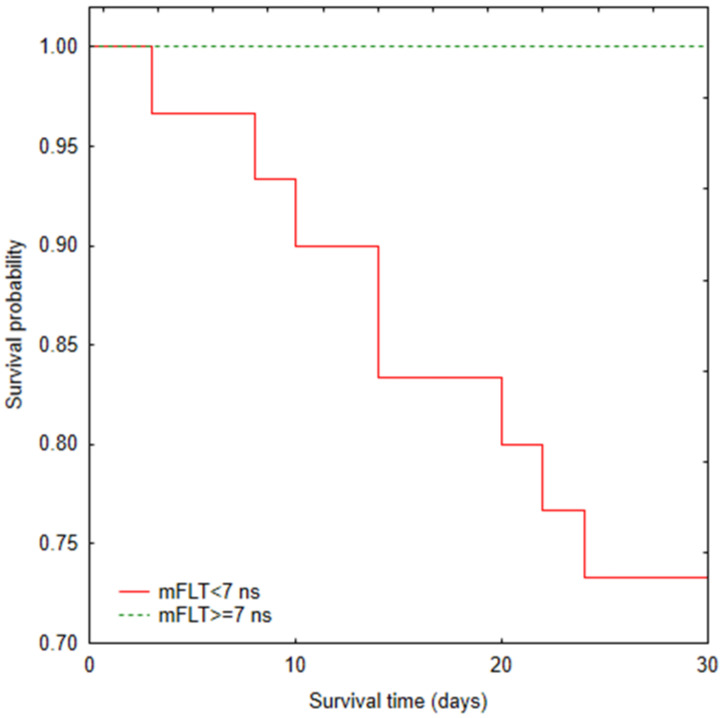
Kaplan–Meier plot for probability of survival for mFLT. Log-rank test *p* = 0.001.

The Kaplan–Meier survival curves (Figure 4) showed significant differences between COVID-19 patients according to mFLT (<7 ns vs. ≥7 ns) for the 30-day mortality variable (*p* = 0.001). There was no culmination of deaths within 30 days. There was also no correlation between mFLT and death time.

### 3.3. A Correlation Study in COVID-19 Patients 

In the presented study, no correlation of mFLT with most clinical parameters was observed. A slight correlation (*p* < 0.05) was observed only for some parameters: WBC (r = 0.28), neutrophils count (r = 0.34), and platelets count (r = 0.39). The mFLT was not dependent on the age and gender of patients. Moreover, prior medical conditions did not appear to affect mFLT. Smoking cigarettes was also not associated with the mFLT. There was also no correlation between the radiological score of computed tomography (HRCT) and the mFLT.

## 4. Discussion

The decrease in mFLT value of plasma may be related with the appearance of additional collagen degradation products in the blood circulation during the process of lung remodeling.

SARS-CoV-2 migrates to the lower respiratory tract and mainly affects type II pneumocytes [31], followed by the secretion of cytokines [32]. The inflammation process is followed by alveolar oedema and hyaline membranes over the damaged alveolar septa [33]. At the end of this process, septal terminal fibrosis appears, characterized by exacerbated proliferation of fibroblasts and excessive deposition of extracellular matrix (ECM) [33,34,35,36]. Collagens I (mature) and III (immature) are the primary components of ECM [35].

Compounds that may have a potential role in an increase of collagen degradation products in blood circulation are MMPs. MMPs degrade the ECM. In fact, following the viral infection by SARS-CoV-2, an ECM breakdown takes place which leads to aggravation of the lung, and provokes progression of lung pathologies [37]. The exacerbated activity of MMPs from neutrophils in acute lung injury (ALI) patients is responsible for the destruction of the alveolar parenchyma. Neutrophils release proteinases, such as metalloproteases MMP-9 and MMP-12, resulting in changes in lung architecture [38,39]. Some researchers observed a higher concentration of MMP-2, 3, and 9 in the blood of COVID-19 patients on admission to the hospital compared to healthy people. The severity of the disease was related to the concentrations of examined MMPs [40,41,42,43,44]. MMP-3 activity is also elevated in ARDS patients, according to Kadry et al. [43]. Furthermore, higher MMP-2 and MMP-9 levels were independent risk factors for mortality in COVID-19 patients [41]. Lerum and co-authors recently reported that persistent pulmonary pathology after COVID-19 is associated with high levels of MMP-9 [42]. Therefore, altered ECM deposition and remodeling appear to represent a key pathological feature of pulmonary fibrosis, and a potential therapeutic target to prevent, delay, or alleviate long-term morbidity and mortality.

The lack of correlation between the HRCT score and mFLT can be explained by the fact that CT scores do not always correspond to increased collagen deposition during severe inflammation. It is difficult to distinguish places of viscous secretions seeping through the pulmonary alveoli accumulations from typical places of fibrosis in the pulmonary parenchyma [45]. Some studies confirmed that CT findings had a weak correlation with collagen amounts in post-mortem lung tissue [46]. Moreover, the microscopic foci of fibrosis can be located in many places that are not visible in CT images. Several studies have shown that the majority of patients with severe COVID-19 have radiographic sequelae of lung disease at mid-term follow-up. In almost all of these patients, fibrosis-like lesions are present as a dominant feature, or alongside non-fibrotic lesions. However, true fibrotic lesions, such as honeycombs, are rarely observed. There is a need for long-term cohort studies to understand the fate of fibrotic-like sequelae in the lung. The time period from the acute phase to the formation of fibrotic lesions in CT is unknown.

The thesis about the appearance of collagen degradation products in the blood is also confirmed by the decreased mFLT observed in patients with bacterial pneumonia without COVID-19 infection. A total of 9 out of 10 patients had a lower mFLT compared to the range in healthy patients. Bacterial pneumonia is well characterized by inflammation and an increased rate of ECM remodeling that results in fibrosis [47].

In addition, in a small sample of patients with idiopathic pulmonary fibrosis it has been shown that the mFLT is reduced compared to healthy subjects. The symptoms of IPF are a little similar to those caused by SARS-CoV-2 [48]. Other studies have shown an increase in both collagen degradation products as well as metalloproteinase activities in the blood of such patients [20,21]. However, the process and causes of fibrosis have not yet been fully explained.

Understanding the course of COVID-19 among hospitalized patients is important for clinicians who are seeking to improve health outcomes in this high-risk population [9,49]. Estimates of mortality rates in patients admitted to hospital with COVID-19 are particularly important for tracking the quality and effectiveness of hospital care [50]. Many researchers revealed that post-mortem lung biopsy showed a higher collagen exposure in tissue [46]. A decrease in mFLT to below 7 ns was an independent risk factor for mortality in our COVID-19 cohort. The mortality rate in COVID-19 hospitalized patients was about 12%. An mFLT below 7 ns (*n* = 30) was associated with a mortality rate of 27%, while no deaths were recorded at the higher levels.

It is still premature to know whether lung changes occur as a temporary response to COVID-19 infection, and if they will spontaneously resolve over time, or if they represent an irreversible pathological feature caused by the viral infection that will persist in surviving patients. Surely, the data collected until now suggest that pulmonary fibrosis persists for many months after recovery from COVID-19 in some patients. Finally, it is still unknown whether the pulmonary fibrosis developed following COVID-19 infection is stable or progressive, especially considering possible genetics, aging, and metabolic risk factors; further investigations are needed [51,52,53]. In our study, some patients still had a reduced mFLT below 7 ns at the next 6-month follow-up. Many researchers also observed abnormal CT results and respiratory parameters (DLCO, TLC, etc.) in many patients 6 months after hospitalization [19,54]. Fluorescence lifetime measurements analysis may allow for quick identification of such patients. In addition, the presented method can be used to track fibrosis progression in these patients. However, it is still uncertain whether the fibrosis-like lesions represent irreversible pulmonary fibrosis. The answer to this question requires studies that involve longer follow-ups as well as larger study groups [55].

## 5. Limitations

This study has some limitations: small sample size in each group, a single-centre donation, and variable time interval of each patient from hospital department admission (up to two days difference) to collection of blood. 

The purpose of the study was to estimate the mortality of patients admitted to the hospital in a mildly severe to moderate state, not demanding invasive mechanical ventilation. For this reason, patients in very severe clinical conditions were excluded from this study.

An unequivocal statement on whether the obtained results reflect actual pulmonary fibrosis requires confirmation, e.g., using lung biopsies and blood collagen degradation products concentration measurements. It is important to point out that this experiment was preliminary, and further investigation in this field of research is crucial. It should also be carefully examined whether other components related to COVID-19 mortality risk can influence the fluorescence lifetime at 450 nm.

## 6. Conclusions

It was demonstrated that the method of detecting collagen degradation products based on time-resolved fluorescence spectroscopy may have significant diagnostic value in the assessment of pulmonary fibrosis. The presented results indicate that a shorter mFLT of plasma samples upon admission to hospital may predict a more severe course of the disease, leading to lung damage, and hence a poorer prognosis. Moreover, the method can be used to identify individuals with post-COVID-19 syndrome who are at high risk of developing pulmonary fibrosis for further diagnoses using highly specific diagnostic tests. Although this hypothesis requires further testing, the numerous benefits of using the fluorescence lifetime measurement encourage consideration of its application in clinical practice. The fluorescence lifetime detection method may also be suitable for clinical screening tests because this method is non-invasive, not time-consuming, and requires only simple pre-treatment of samples without using additional reagents.

## Figures and Tables

**Table 1:** Baseline demographic and disease characteristics.

	Study Group—COVID-19 (C)	Study Group—COVID-19 after 6 Months (C-6M)	Study Group [Bacterial Pneumonia; Non-COVID-19] (BP)	Study Group [Idiopathic Pulmonary Fibrosis] (IPF)	Control Group (H)
Number	66	30	10	9	15
Mean age (range) [years]	62.3 (25–98)	60.6 (30–87)	62.4 (41–90)	68.3 (62–87)	40.8 (25–61)
Gender					
Women	13 (20%)	5 (17%)	1 (10%)	3 (33%)	12 (80%)
Men	53 (80%)	25 (83%)	9 (90%)	6 (67%)	3 (20%)
Smoking					
Yes	16 (24%)	5 (17%)	7 (70%)	4 (44%)	3 (20%)
No	50 (76%)	25 (83%)	3 (30%)	5 (56%)	12 (80%)
Common symptoms:					
Dyspnoea	53 (80%)		6 (60%)		
Cough	51 (77%)		3 (30%)		
Fever	60 (91%)		1 (10%)		
Myalgia	21 (32%)		1 (10%)		
Changes in the sense of smell and/or taste	11 (16%)		0		
Common comorbidities:					
Cardiovascular diseases	34 (51%)	4 (13%)	7 (70%)		
Type 2 diabetes	14 (21%)	3 (10%)	3 (30%)		
Previous lung diseases	6 (9%)	2 (7%)	4 (40%)		
Cancer	4 (6%)	2 (7%)	3 (30%)		

## Data Availability

The data presented in this study are available upon request from the corresponding author.
